# Simulating macroevolutionary trends and open-ended evolution with a novel mechanistic multi-level approach

**DOI:** 10.1371/journal.pone.0335033

**Published:** 2025-11-06

**Authors:** Roberto Latorre, Miguel Brun-Usan, Gloria Fernández-Lázaro

**Affiliations:** 1 Grupo de Neurocomputación Biológica, Dpto. de Ingeniería Informática, Escuela Politécnica Superior, Universidad Autónoma de Madrid, Madrid, Spain; 2 Dpto. de Biología, Unidad de Paleontología, Facultad de Ciencias and Centre for the Integration in Paleobiology (CIPb-UAM), Universidad Autónoma de Madrid, Madrid, Spain; 3 Centro Andaluz de Biología del Desarrollo (CABD), Universidad Pablo de Olavide-CSIC, Seville, Spain; 4 Dpto. de Psicología Biológica y de la Salud, Facultad de Psicología, Universidad Autónoma de Madrid, Madrid, Spain; National Cheng Kung University, TAIWAN

## Abstract

Microevolution and macroevolution describe evolutionary change at different scales: short-term genetic and phenotypic variation within populations, and long-term patterns of diversification and extinction. Despite their interconnected nature, they have often been studied separately, and the reciprocal causal links between them remain poorly understood due to their operation on different timescales and the complexity of the processes involved, making mechanistic approaches particularly challenging. To bridge this operational gap, we introduce a novel bottom-up, process-based computational framework that integrates genotype-to-phenotype mapping, fitness evaluation under environmental constraints, and biotic interactions shaping ecological niches and adaptive pressures, while incorporating lower-level mechanisms such as mutation, gene flow, and gene-pool expansion through stochastic duplication of genes. Its modular design accommodates diverse microevolutionary mechanisms to study the emergence of large-scale eco-evolutionary patterns from explicit individual-level processes.

The framework allows addressing research questions ranging from the formation of spatiotemporal biodiversity patterns to the role of eco-evolutionary feedbacks in macroevolution. It provides an open-ended platform that serves both as a theoretical tool for testing evolutionary hypotheses and as a flexible environment for exploratory simulations. To illustrate its heuristic potential, we present proof-of-concept simulations under biologically plausible conditions that reproduce multiple well-documented macroevolutionary patterns–such as biphasic diversification, saturating and exponential-like biodiversity trends, speciation–extinction correlations, species duration distributions, and niche structuring–as emergent phenomena. Beyond reproducing patterns, the simulations reveal underlying mechanisms, including trial-and-error dynamics in long-term adaptation, high species turnover maintaining biodiversity equilibrium, and self-organized niche occupancy.

These findings establish the framework as a versatile tool for investigating the complex interplay of ecological and evolutionary forces shaping biodiversity. By capturing emergent dynamics from mechanistic microevolutionary processes without imposing predefined constraints, the model provides a unique perspective on long-term evolutionary change, contributing to a broader theoretical toolkit for studying macroevolutionary patterns under controlled conditions. Future extensions could assess how variations in environmental dynamics, genomic architecture, or species interactions influence evolutionary trajectories, refining our understanding of biodiversity evolution.

## Introduction

The ever-changing environmental and ecological conditions faced by organisms drive evolutionary processes, shaping biodiversity over time [1]. Adaptation to these dynamic conditions determines species’ persistence or extinction [2], highlighting the need to unravel the eco-evolutionary mechanisms underlying long-term biodiversity dynamics. Evolutionary change emerges from the interplay of multiple factors–e.g., genetic variation, ecological interactions, environmental shifts, phenotypic plasticity, developmental constraints, and niche construction–acting across biological and temporal scales to shape eco-evolutionary systems [3]. Understanding how the reciprocal interaction between these mechanisms impacts long-term biodiversity patterns remains a central challenge in evolutionary biology [4–8]. Despite substantial progress, fundamental questions about the origins, diversification, and distribution of life persist, largely due to the complexity of multiscale evolutionary processes [9–15].

To address the complexity of these entangled, multicausal processes, evolutionary research traditionally distinguishes between two broad categories: *microevolution*, which examines genetic, developmental, and phenotypic changes within populations over short timescales, and *macroevolution*, which focuses on long-term patterns of diversity, speciation, and extinction “above the species level” [16,17]. Although historically treated as separate fields [10,18–20], their interdependence is now widely recognized as fundamental to understanding biodiversity dynamics [17,21,22]. However, establishing causal links between microevolutionary processes and macroevolutionary outcomes remains challenging. Traditional approaches to macroevolution, such as fossil record analyses [23,24] and phylogenetic reconstructions [25–28], provide invaluable insights into large-scale biodiversity trends but offer limited resolution on how short-term eco-evolutionary mechanisms shape long-term diversification and extinction patterns [29–34]. Addressing this gap requires complementary mechanism-based approaches capable of explicitly modeling interactions across biological and temporal scales [35].

In this context, computational models have emerged as powerful tools for integrating micro- and macroevolutionary processes [36–40]. By simulating eco-evolutionary dynamics under controlled conditions, where key parameters and interactions can be systematically manipulated, these *in-silico* approaches allow researchers to explore how short-term evolutionary mechanisms scale up to shape long-term biodiversity patterns [41–44]. Beyond reproducing observed patterns, simulations provide a means to test mechanistic hypotheses, investigate causal relationships, and generate novel predictions about emergent evolutionary dynamics that may be difficult or even impossible to infer directly from empirical data [13,45,46].

Existing macroevolutionary computational models vary widely in scope, complexity, and underlying assumptions. Taking inspiration on the fact that the balance between speciation and extinction rates shapes macroevolutionary biodiversity patterns [7,47,48], traditional *top-down* approaches, such as birth-death models of diversification [25,49–51] or species network evolution models [52–55], rely on simplifying assumptions to capture large-scale biodiversity patterns. While these methods provide valuable insights into macroevolutionary dynamics, they do not explicitly implement the underlying eco-evolutionary processes driving evolution. In response to these limitations, generative *bottom-up* approaches have gained increasing attention by integrating mechanistic eco-evolutionary processes into macroevolutionary models [56]. These efforts span a spectrum of methodologies, such as pluralistic models that emphasize species interactions, trait evolution, and environmental dynamics [57,58]; process-explicit simulations of macroecological and biogeographical patterns [38,44,59,60]; models that explore the occupation of morphospace and phenotypic disparity over evolutionary time [61,62]; or computational tools modeling genetically explicit evolutionary dynamics in changing environments [63,64]. Some of these approaches are designed to test specific evolutionary hypotheses, while others aim to provide more general-purpose solutions for exploring broad evolutionary scenarios.

Despite these advances, achieving a comprehensive integration of eco-evolutionary feedbacks across biological levels–from genotypes to ecosystems–is still an outstanding challenge, particularly in models seeking to balance flexibility, mechanistic depth, and computational tractability. To address this, a variety of computational techniques have been employed, e.g., agent-based modeling [65], cellular automata [66], network analysis [63], and differential equation systems [67]. While each of these approaches has strengths, they also present inherent limitations in scalability, generality, or the ability to explicitly model genotype-phenotype interactions.

In this paper, we introduce a framework built upon this growing body of general-purpose evolutionary models, although it substantially departs in many aspects from previous approaches, resulting in a qualitatively distinct simulation platform. At its core, it is inspired by *Grammatical Evolution* (GE) [68–70], a versatile computational technique that explicitly links genotype to phenotype through a flexible generative algorithm. This property, among others, makes GE particularly well-suited for capturing the emergence of complex traits and simulating long-term eco-evolutionary dynamics. Nevertheless, despite its widespread application in other fields (e.g., see [71–73]), its potential for modeling evolutionary processes in biological systems remains largely unexplored.

In our framework, evolution unfolds at the population level, with populations treated as the primary units of evolution. Each population carries a heritable genotype, and a GE-based genotype-to-phenotype mapping (GPM) allows a nonlinear transformation from genotype to phenotype, where small changes in the genetic substrate can lead to significant phenotypic variation or shifts in gene functionality. This effectively captures the complexity of real-world GPMs [74,75], facilitating the evolution of complex traits and adaptive novelties without imposing predefined developmental or adaptive constraints. Evolutionary dynamics take place within an explicit two-dimensional dynamic environment structured into regions that change over time. These regions provide the ecological context in which populations thrive, coevolve, and migrate, adapting to both abiotic and biotic conditions. By incorporating elements of both the *Court Jester* [76] and *Red Queen* [77] approaches, the framework captures a spectrum of eco-evolutionary scenarios where species survival depends on a dynamic interplay of ecological constraints, competition, and environmental fluctuations [78,79]. In this scenario, fitness is assessed contextually, prioritizing functionality and ecological performance over direct measurements of phenotypic traits or their genetic substrate. This context-dependent evaluation drives asymmetric competition and plays a key role in niche structuring.

A key strength of our framework lies in its process-explicit design, flexibility and open-ended nature [80]. By customizing initial conditions, which eco-evolutionary processes are simulated, and their corresponding parameters, rules, and functions, the model can generate a broad range of evolutionary scenarios, from stable equilibrium-like dynamics to highly dynamic, innovation-driven landscapes. This flexibility allows for both detailed, hypothesis-driven simulations and broader exploratory studies aimed at understanding biodiversity evolution in complex, dynamic environments. Moreover, unlike models where evolutionary trajectories are predefined or limited to a few possible outcomes [81,82], here evolutionary innovations emerge naturally from the interplay of genetic architecture, selection, and ecological dynamics, enabling macro-scale patterns to emerge from micro-scale processes and supporting a continuously expanding evolutionary space.

To sum up, the primary aim of this study is to introduce and describe a novel computational framework for simulating eco-evolutionary dynamics across temporal and biological scales. Rather than addressing a specific evolutionary question or testing predefined hypotheses, our goal is to present the structure, rationale, and heuristic potential of the model, emphasizing its flexibility, generative capacity, and conceptual coherence as a foundation for future theoretical developments and computational applications in evolutionary biology. With it, we try to demonstrate how complex macroevolutionary patterns can emerge naturally from fine-scale, population-level eco-evolutionary interactions by presenting proof-of-concept simulations conducted under biologically plausible conditions. These simulations provide insights into the mechanisms driving biodiversity evolution, highlighting the role of continuous species turnover, adaptive diversification, ecological structuring, and trial-and-error dynamics in shaping long-term evolutionary trajectories. A comprehensive empirical validation, including specific case studies, parameter benchmarking, and comparisons with existing models, is beyond the scope of this initial work and will be addressed in future studies.

## Methods

Our modular simulation framework integrates genetic, phenotypic, and ecological processes into a general eco-evolutionary model. Evolutionary dynamics are modeled at the level of populations, which interact with changing environments and with other co-evolving populations, both within and across species. The simulation proceeds iteratively from an initial state, which is updated at each time step through a sequence of eco-evolutionary processes, including genetic variation, niche adaptation, migration, ecological interactions, and extinction, among others. These processes are predominantly stochastic, although deterministic or context-dependent rules can be implemented as needed, allowing for flexible and extensible implementation of hypothesis-driven experimentation within an open-ended evolutionary framework.

Time steps in the simulation are abstract units rather than direct equivalents of biological time (e.g., generations or years). They are conceptualized as intervals in which measurable population-level changes (e.g., speciation, extinction, or adaptive shifts) may occur, which makes it possible to study how large-scale evolutionary patterns emerge without tying the model to a specific temporal scale.

**Fig 1:**
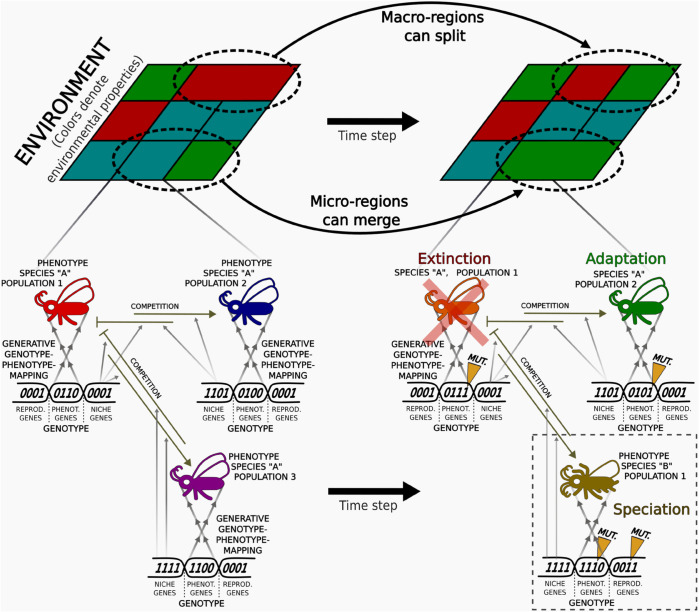
Conceptual representation of the main components of the proposed model. Schematic representation of an initial 3×3 square-shaped landscape. Environmental dynamics influence regional properties (illustrated by different colors) and produce large-scale geographical variations that affect genetic exchange, migration, and speciation. Three populations of a hypothetical insect species coevolve within a given region, each exhibiting distinct genotypes and phenotypes that determine their adaptability to regional properties. Genetic variation, induced by mutation and gene flow, drives the emergence of phenotypic innovations (e.g., color changes or presence of legs or antennae) via changes in phenotypic genes, speciation events via changes in reproductive genes, and ecological interactions via changes in niche genes. For further details, see the main text.

The two following subsections describe, respectively, the model’s core components and eco-evolutionary processes (supported by a conceptual diagram in Fig 1), and the parameters used in our proof-of-concept simulations. Additionally, a simplified, resource-constrained implementation of the framework is publicly available as an interactive web application at https://ecoevoframeworkdemo.streamlit.app/, allowing readers to run limited simulations and explore in real time the effect of certain processes and parameters.

### Framework description

#### Environmental dynamics.

The *environment* serves as the spatiotemporal context in which populations and species evolve (Fig 1, top). Initially, it is divided into $d_{1}\;\times \;d_{2}$ discrete *regions*, organized in a two-dimensional grid with periodic boundary conditions to ensure continuity at the edges. Over time, the number and configuration of these regions can change dynamically due to environmental processes.

Each region is characterized by a set of $n_{env}$ bit sequences representing its *environmental properties*. These act as abstract proxies for abiotic and biotic factors relevant to survival, adaptation, and dispersal–such as temperature, resource availability, or oxygen levels (generically represented as regional color patterns in Fig 1). Environmental properties evolve stochastically in each iteration with a per-region probability $p_{region_{change}}$, introducing temporal variability at the bit level to simulate dynamic environmental conditions.

To model large-scale environmental dynamics analogous to geological or climatic events (e.g., shifts in habitat connectivity, or formation of new ecological barriers), the framework includes two structural mechanisms:

- *Region merging:* Adjacent regions can combine into macro-regions with probability $p_{env_{merge}}$, inheriting a random combination of the environmental properties of the original regions.
- *Region splitting:* Macro-regions can fragment into smaller adjacent regions with probability $p_{env_{split}}$, retaining the environmental properties of the original region.

These mechanisms introduce spatial and temporal heterogeneity into the environmental landscape by modulating the degree and rate of habitat restructuring, thereby shaping the conditions under which eco-evolutionary processes such as adaptation, migration, and speciation unfold. For instance, increasing $p_{region_{change}}$ intensifies temporal variability within regions, potentially driving higher rates of adaptation and extinction. Likewise, higher values of $p_{env_{merge}}$ promote regional homogenization and connectivity, while elevated $p_{env_{split}}$ values enhance habitat fragmentation and isolation—both of which influence dispersal, gene flow, and the potential for speciation.

#### Populations as units of evolution.

Populations are treated as the fundamental units of evolutionary change. While individual-based models offer fine-grained biological realism, they become computationally intractable when scaled to macroevolutionary levels [42]. Conversely, species-level abstractions often overlook key within-species dynamics, such as local adaptation, lineage diversification, or population-specific ecological interactions. Modeling evolution at the population level provides an effective balance between biological detail and computational feasibility.

*Populations* are conceptualized as cohesive evolutionary entities that, under a strong-selection, weak-mutation regime [83], exhibit low internal genetic variability. Each population is thus characterized by a predominant *featured genotype*, which encapsulates the cumulative genetic, phenotypic, and ecological characteristics of its members. While this population-genotype-based abstraction assumes a dominant genotype within each population, it allows for substantial genetic variability across populations belonging to the same species. For example, in Fig 1, the red, blue, and purple populations initially belong to the same insect species but display distinct genotypes and phenotypes, enabling differential adaptation to different ecological contexts.

At a higher level of organization, *species* are defined in accordance with the biological species concept [84] as groups of interbreeding populations that share a common evolutionary history and are reproductively isolated from others such groups.

Population genotypes are modeled as variable-length bit sequences, structured into three abstract gene categories: (i) *phenotypic genes*, which encode functional traits; (ii) *reproductive genes*, which determine mating compatibility and govern speciation; and (iii) *niche genes*, which mediate ecological interactions among populations. Although these components are not literal molecular representations of biological material, they offer a mechanistic formalism for modeling the heritable basis of eco-evolutionary processes.

#### Mechanisms for genetic variation.

Genetic variation in the simulation framework arises through two primary mechanisms:

- *Mutation* introduces stochastic bit-level modifications to the genotypes of populations in each iteration. Independent mutation rates are assigned to phenotypic ($p_{mut_{p}}$), reproductive ($p_{mut_{r}}$), and niche genes ($p_{mut_{n}}$), allowing differential control over the evolutionary dynamics of each gene category. Niche genes of type *n* do not mutate directly but are modified through macroevolutionary processes (see “Niche differentiation”). Typically, higher mutation rates must be assigned to phenotypic genes to reflect their greater role in short-term adaptive responses, whereas reproductive and niche genes mutate more slowly to simulate the gradual accumulation of barriers to interbreeding and ecological niche shifts.
- *Gene flow* allows for the exchange of genetic material between coexisting populations of the same species, while preserving the positional integrity within the genome. This process is governed by parameters $p_{flow_{p}}$, $p_{flow_{r}}$, and $p_{flow_{n}}$, which regulate the exchange of phenotypic, reproductive, and niche genes, respectively. Although primarily designed to simulate sexual reproduction, asexual reproduction can be approximated by setting these probabilities to zero.

Together, these mechanisms generate genetic diversity that underlies phenotypic innovation and drives key evolutionary processes such as speciation and niche adaptation (see subsequent sections for details). Increasing mutation or gene flow rates can accelerate the pace of evolutionary change, while lower values contribute to genetic stability and lineage persistence.

In addition, the framework includes genome expansion, allowing populations to increase the number of genes within each genomic segment. By default, this process is modeled as stochastic duplication of existing genes and is governed by the parameters $p_{exp_{p}}$, $p_{exp_{r}}$, and $p_{exp_{n}}$ for phenotypic, reproductive, and niche genes, respectively. Once duplicated, the new gene diverges from the original via subsequent mutation and gene flow, and the resulting configuration is heritable along lineages. Although the mechanism abstracts from molecular detail, it captures the functional consequences of genome growth in an open-ended evolutionary context by modifying the genomic substrate on which selection acts. Genome expansion itself does not trigger speciation unless it contributes to reproductive isolation (see “Speciation”). Accordingly, higher expansion rates *a priori* increase opportunities for phenotypic complexity, ecological adaptation, and diversification; whereas lower rates promote genomic stability and reduce the likelihood of large-scale functional shifts.

#### Genotype-to-phenotype mapping.

To capture the diversity and complexity of phenotypic traits observed in natural populations, the framework employs a generative genotype-to-phenotype mapping (GPM) that connects the genetic substrate of populations to their expressed phenotypes. Specifically, the *phenotypic genes* within each population’s genotype (collectively referred to as the phenotypic segment) encode a set of functional traits abstracting key biological characteristics relevant to adaptation and niche differentiation (e.g., color, number of legs, or presence of antennae in Fig 1).

The GPM is implemented using a grammar-based evolutionary algorithm that uses the information encoded in the phenotypic segment to generate a series of mathematical expressions, each corresponding to a distinct *phenotypic trait*. This process is independent of genome size, naturally enabling consistent trait derivation across populations with varying numbers of phenotypic genes. Our *GE-based GPM* relies on a context-free grammar that defines a set of generative (or production) rules, which recursively expand an initial non-terminal symbol until a terminal expression is produced (S1 Fig). Unlike alternative generative models that depend on finely tuned parameters, complex agent interactions, or high-order statistical correlations [82,85], this approach allows complexity and nonlinearity to emerge intrinsically from the mapping process.

Production rules can specify multiple derivation options for each symbol, allowing for diverse trait outcomes. Here, *codons*, defined as fixed-length bit sequences located within the phenotypic segment, play a critical role since they serve as positional markers that guide the choice of production rules during mapping. Their size is determined by the maximum number of derivation options in the grammar (e.g., two bits for up to four options). A single codon can span bits from multiple phenotypic genes, and multiple codons may be embedded within a single gene, enhancing both flexibility and modularity in trait generation. Not all phenotypic genes are expressed: only codons used in the derivation process (highlighted in green in S1 Fig) influence the resulting trait, mirroring selective activation patterns analogous to regulatory dynamics in biological systems.

Two key features of the GE-based GPM promote phenotypic diversity and adaptability. First, its iterative nature allows minor genetic changes, such as a single codon mutation, to produce substantial phenotypic shifts. This non-linearity facilitates the generation of a broad spectrum of traits, mimicking how genetic information in biological systems regulates phenotypic traits. For instance, variations in the phenotypic segments of populations in Fig 1 lead to color changes in *Population 2* of *Species A* and the emergence of additional legs or antennae in *Population 1* of *Species B*. Second, the circular architecture of the phenotypic segment permits codon reuse during the mapping process, significantly amplifying the phenotypic space accessible from compact genotypes. This property mirrors the modular reuse of genetic components in biological systems and enhances the generative capacity of the model.

Together, these features mitigate limitations common to simpler GPMs, such as reduced variability in small genotypes or oversimplifications leading to spurious evolutionary convergence (homoplasy). The GE-based GPM thus provides a robust and flexible mechanism for simulating open-ended phenotypic diversification and evolutionary innovation across extended timescales.

#### Emergence of new populations and migration.

The emergence, diversification, and dispersal of populations are fundamental drivers of eco-evolutionary dynamics in the framework, as biodiversity arises not from explicit species-level processes but from the accumulation and interaction of population-level events (see next section). These processes introduce not only spatial and genetic structure into evolving lineages but also generate new ecological interactions, reshaping local community composition and the broader ecological network. As populations of different species coevolve, they foster local adaptation, ecological divergence, and, ultimately, speciation.

New populations arise through probabilistic splitting events, in which an existing population divides into two subpopulations. This process is governed by a population-specific splitting probability ($p_{pop_{split}}$), which determines the likelihood of such events occurring over time. When a split occurs, the new population may migrate to a neighboring region with probability $p_{pop_{mig}}$, provided adjacent space is available; otherwise, it remains within the same region, coexisting with its parent population.

Higher values of $p_{pop_{split}}$ increase the frequency of population divergence events, *a priori* promoting lineage diversification and spatial expansion. Similarly, increasing $p_{pop_{mig}}$ enhances the dispersal potential of populations, facilitating colonization of new habitats and increasing opportunities for ecological differentiation. However, it is the balance between these two processes what ultimately shapes lineage dynamics. For instance, frequent splitting combined with limited migration can lead to regional coexistence of multiple populations from the same species, fostering both phenotypic diversity and species persistence. Conversely, low rates of both processes may result in evolutionary stasis or extinction due to limited adaptability.

#### Speciation.

The framework adopts the widely accepted view that genetic divergence is the primary driver of speciation [84,86,87]. New species emerge when reproductively compatible populations accumulate sufficient genetic differences to prevent successful interbreeding. Rather than being explicitly programmed, this process arises naturally from underlying eco-evolutionary dynamics involving genetic variability and population-level diversification.

To evaluate interbreeding compatibility, the model focuses exclusively on *reproductive genes*, i.e., the reproductive segment of each population’s genotype. *Speciation* is defined as an emergent and unidirectional process resulting from the gradual accumulation of mutations in this segment. Once two populations become reproductively isolated, speciation is considered irreversible, even if subsequent genetic changes reduce their reproductive distance. Notably, this definition is independent of phenotypic differentiation, allowing for cryptic speciation events within the model.

Reproductive distance between populations is quantified using either normalized Hamming distance or absolute numerical difference. The former measures the proportion of differing bits in the reproductive segment, giving equal weight to all mutations. The latter emphasizes mutations in the most significant bits, effectively weighting genetic changes by positional relevance. Importantly, genome expansion does not disrupt comparability between populations, as both distance measures remain valid and comparable regardless of segment length, provided that bit ordering is consistent. When the reproductive distance between two populations exceeds a predefined threshold (*th*_*speciation*_), they are classified as distinct species. For example, in Fig 1, mutations in the reproductive genes of *Population 3* of *Species A* result in the emergence of reproductive barriers and the formation of *Species B*.

The threshold parameter *th*_*speciation*_ governs the sensitivity of the model to reproductive divergence. Lower values of this threshold facilitate rapid speciation and promote lineage diversification, whereas higher values impose stricter conditions for reproductive isolation, favoring cohesion within species and delaying speciation events.

#### Fitness evaluation.

The *fitness* of a population, conceptualized as its capacity to cope with local environmental conditions, influences its probability of survival and long-term persistence. Fitness values range from 0 to 1 and are determined by a mathematical function (*fitness function*) that, evaluated at each iteration, quantifies the compatibility between a population’s phenotype and the evolving environmental properties of the region it inhabits. A higher degree of alignment between trait values and environmental conditions results in higher fitness scores. In the illustrative example of Fig 1, assuming that fitness depends solely on the match between the phenotypic trait *color* and the environmental property *color*, populations occupying similarly colored regions (e.g., the blue and purple populations in a cyan-green region) exhibit higher fitness than poorly matched populations (e.g., the red population).

To capture diverse adaptive strategies within a single simulation, each population is assigned a characteristic fitness function, generated using a grammar-based approach analogous to the GE-based GPM described earlier. In this case, however, the derivation path is selected stochastically during population initialization rather than being genetically encoded. The grammar includes standard mathematical primitives such as polynomials, exponentials, logarithms, and trigonometric functions, which can be combined to build expressions that evaluate the compatibility between one or more phenotypic traits and environmental properties. This generative approach enables the simulation of a wide range of phenotype-environment relationships without imposing predefined selection constraints.

As a result, fitness functions can define linear or nonlinear relationships, include threshold conditions, or represent complex trade-offs across multiple traits. Depending on their structure, some fitness functions require populations to perform adequately across multiple dimensions to achieve acceptable fitness values, while others depend on precise trait-environment matches along a narrower axis, favoring populations that exhibit sharp adaptation to specific conditions.

The outcomes of fitness evaluation are directly linked to one of the extinction routes modeled in the framework, as detailed in the “Extinction” section. Populations with persistently low fitness values are more likely to go extinct due to environmental mismatch.

#### Niche differentiation.

**Ecological niche**. Following classical niche theory [88], ecological niches are defined as the sets of abiotic and biotic conditions under which a population can persist and reproduce. To model them, the framework adopts a widely used formalization [89] in which three parameters characterize each population’s ecological profile along an abstract gradient: the actual location (*niche position*, *n*), the preferred conditions (*niche optimum*, *c*), and the tolerance to environmental variation (*niche range*, *r*). These parameters are encoded in three types of niche genes (*n*, *c*, and *r*) within the population’s genotype. While abstract, these genes provide a flexible substrate for encoding eco-evolutionary interactions defining two essential ecological properties:

- The *fundamental niche width*, defined by the interval [c−r,c+r], represents the potential range of conditions under which a population could survive.
- The *realized niche width*, defined by [n−r,n+r], reflects the actual conditions experienced by the population at a given point in time.

This distinction enables the model to separate intrinsic ecological potential from realized ecological occupancy, a key concept in niche theory. At the same time, genome expansion in the niche segment increases the number of independent niche dimensions a population can access, enabling not only more flexible patterns of niche overlap, but also potential adaptive escape routes into unoccupied ecological space.

**Ecological relationships**. Niche parameters mediate ecological interactions among populations. Each region maintains a dynamic ecological network in which populations are nodes, and directed, weighted links represent ecological relationships. These are classified as either competitive or non-competitive and are updated at each iteration.

- *Competitive interactions* arise when populations within the same region exhibit overlapping realized niches, leading to the automatic establishment of bidirectional negative links.
- *Non-competitive interactions* may occur between populations whose realized niches do not overlap. These interactions can be *facilitative* (positive) or *inhibitory* (negative), encompassing ecological relationships such as mutualism, commensalism, amensalism, parasitism, or pollination. Unlike competitive interactions, their formation and removal are stochastic, governed by the probabilities $p_{rel_{add}}$ and $p_{rel_{del}}$, respectively. The nature and direction of each interaction follow allometric principles derived from niche models that correlate niche position with organism size [89,90], whereby larger organisms (with higher *n* values) tend to facilitate smaller ones–e.g., trees shading understory plants–while smaller organisms more often exert inhibitory effects on larger ones–e.g., parasites affecting hosts.

Interaction strengths are determined dynamically based on the ecological context, as quantified by the net ecological impact (*NEI*) metric described in the following section.

**Niche evolution and adaptive shifts**. Niche dynamics in the framework emerge through two distinct pathways: a micro- and a macroevolutionary pathway. In the microevolutionary pathway, governed by genetic variation, changes in niche optimum (*c*) and niche range (*r*) genes alter the population’s fundamental niche over time, modulating its potential ecological tolerances and interactions. In contrast, niche position genes (*n*) remain genetically immutable and only change through macroevolutionary shifts triggered by ecological interactions.

The macroevolutionary pathway is activated when a population experiences sustained negative ecological pressure. This pressure is quantified through the *NEI*, a dynamic metric that integrates the effects of both competitive and non-competitive interactions:

$$NEI_{A}=\sum_{i\neq A}\left[\left(L_{A,i}\cdot \left(1-F_{A,i}\right)\right)+W_{A,i}\right]$$ (1)

Here, *L*_*A*,*i*_ denotes the degree of niche overlap between populations *A* and *i*, *F*_*A*,*i*_ the fitness difference between them (modulating the impact of competition), and *W*_*A*,*i*_ the cumulative effect of facilitative and inhibitory interactions. Positive *NEI* values indicate favorable ecological conditions and integration within the community, while negative values reflect ecological stress.

When a population’s *NEI* drops below a given threshold ($th_{niche_{shift}}$), an adaptive shift is triggered, involving a displacement of its niche position (*n*), an adjustment of its niche range (*r*), or both, by ±1 unit. This threshold modulates the population’s responsiveness to unfavorable ecological interactions: higher values delay the response, allowing populations to remain under stronger competitive or inhibitory pressures without immediate reaction, while lower values increase sensitivity, promoting earlier shifts as an adaptive strategy to reduce conflict. These changes aim at mitigating ecological stress and reconfiguring biotic interactions, ultimately improving the population’s chances of long-term persistence. Further details and examples are provided in S1 Text.

#### Extinction.

*Species extinction* is modeled as an irreversible, emergent phenomenon, resulting from the cumulative collapse of all constituent populations. Each population’s fate is shaped by its capacity to adapt and persist under dynamic environmental and ecological conditions, which together generate multiple interacting pressures, including environmental mismatches, ecological conflicts, and niche instability. To capture this diversity of extinction drivers, three complementary extinction routes are implemented, each representing distinct but interconnected biological processes:

- *Fitness-driven extinction*. This route models population collapse due to persistent maladaptation to local environmental conditions. When a population’s fitness remains below a minimum threshold ($th_{ext_{fit}}$), it is considered no longer viable and is removed from the simulation. In the example of Fig 1, *Population 1* of *Species 1*, poorly adapted to its environmental context, goes extinct in the final state, whereas *Population 2* persists due to higher fitness. This mechanism reflects classical ecological theory [2,91–93], where selection gradually eliminates poorly adapted populations, especially under abrupt or large-scale environmental change. Increasing $th_{ext_{fit}}$ intensifies selection by requiring higher phenotype-environment compatibility for population persistence.
- *Ecological-interactions-driven extinction*. This fitness-independent route emphasizes the role of ecological pressures–such as sustained competition, parasitism, or inhibitory interactions–in destabilizing population viability [94–97]. When the *NEI* of a population drops below a collapse threshold ($th_{ext_{rel}}$), the ecological context is considered too hostile to sustain the population, leading to its extinction even if the population exhibits high intrinsic fitness. This threshold regulates a population’s tolerance to cumulative ecological pressure: lower values represent higher resilience, while higher values cause collapse under milder negative interactions.
- *Niche mismatch extinction*. This route captures scenarios where disruptive genetic variation or maladaptive shifts–both resulting from dynamics not shaped by adaptive guidance–lead populations into unsuitable niche configurations. Thus, when a population’s realized niche position (*n*) falls outside its fundamental ecological tolerance range ([c−r,c+r]), the population becomes ecologically unviable and is therefore removed from the simulation.

### Parameter setup and rationale for simulation design

The simulations presented in this study were not intended to exhaustively examine the high-dimensional parameter space of the framework. Instead, we explored different representative emergent evolutionary phenomena, spanning different temporal and geographic scales, to illustrate the framework’s capacity to produce plausible evolutionary dynamics under interpretable biological conditions. In line with general principles for developing large-scale eco-evolutionary models that balance computational feasibility with biological realism [42,98], the configuration was selected through prior systematic testing to identify parameter combinations that promote ecological structure, evolutionary innovation, and lineage persistence, while avoiding unrealistic collapse or unchecked proliferation [91]. The aim was to simulate conditions under which populations experience meaningful adaptive challenges across spatial and temporal scales, enabling the emergence of complex biodiversity patterns without requiring parameter fine-tuning or calibration to specific empirical data.

Table 1 lists all parameters used in our proof-of-concept simulations. Below, we briefly outline the biological interpretation and rationale behind key parameter categories, consistent with the process-level descriptions provided earlier. To preserve a degree of open-endedness in system dynamics, no artificial limits–either local or global–were imposed on the number of populations, species or ecological configurations that could emerge during simulations. Fitness functions were also generated randomly to avoid any predetermined selection pressure. The only structural constraints derive from the genotype architecture itself, which defines the resolution of ecological differentiation among populations without restricting their evolutionary potential (see below).

**Table 1:** Parameter values used in the proof-of-concept simulations.

Parameter	Description	Scope	Value
**Environment and its dynamics**			
$d_{1}\times d_{2}$	Environment dimensions (number of regions)	Global	1000×1000
$n_{env}$	Number of environmental properties per region	Global	24
*size*_*property*_	Number of bits per environmental property	Global	8
$p_{region_{change}}$	Probability of change in an environmental property per region	Global	U(0.005,0.025)
$p_{env_{merge}}$	Probability of region merging	Global	0.0 (process disabled)
$p_{env_{split}}$	Probability of region splitting	Global	0.0 (process disabled)
**Genotype architecture**			
$n_{gen_{p}}$	Default number of phenotypic genes per population	Species	36
$n_{gen_{r}}$	Default number of reproductive genes per population	Species	2
$n_{gen_{n}}$	Default number of niche genes per population	Species	1
*size*_*gene*_	Number of bits per gene	Global	8
**Genetic variation**			
$p_{mut_{p}}$	Rate of mutation for phenotypic genes	Population	U(0.0,0.0005)
$p_{mut_{r}}$	Rate of mutation for reproductive genes	Population	U(0.0,0.0001)
$p_{mut_{n}}$	Rate of mutation for niche genes (excluding *n*)	Population	U(0.0,0.00001)
$p_{flow_{p}}$	Probability of gene flow for phenotypic genes	Population	$𝒩(p_{flow_{f_{A}}},\;\sigma =0.001)$
$p_{flow_{r}}$	Probability of gene flow for reproductive genes	Population	$𝒩(p_{flow_{r_{A}}},\;\sigma =0.001)$
$p_{flow_{n}}$	Probability of gene flow for niche genes	Population	$𝒩(p_{flow_{n_{A}}},\;\sigma =0.001)$
$p_{exp_{p}}$	Probability of phenotypic segment expansion	Species	$5\cdot 10^{-7}$
$p_{exp_{r}}$	Probability of reproductive segment expansion	Species	$1\cdot 10^{-7}$
$p_{exp_{n}}$	Probability of niche segment expansion	Species	0.0 (process disabled)
**Population dynamics**			
$p_{pop_{split}}$	Probability of population splitting into subpopulations (originating from population A)	Population	$𝒩(p_{A_{split}},\sigma =0.0025)$
$p_{pop_{mig}}$	Probability of migration to adjacent regions after population splitting	Population	$𝒩(p_{A_{mig}},\sigma =0.0025)$
**Speciation and extinction**			
*th*_*speciation*_	Threshold for reproductive Hamming distance	Species	$n_{gen_{r}}\cdot size_{gene}\cdot U(0.5,\;0.9)$
$th_{ext_{fit}}$	Minimum fitness threshold for fitness-driven extinction	Species	0.10
$th_{ext_{rel}}$	Threshold for ecological-interactions-driven extinction (negative *NEI*)	Species	*U*(–1.0,–0.25)
—	Niche-mismatch extinction process	Global	enabled

Parameters with a global scope are fixed throughout the entire simulation, whereas species- and population-level parameters are assigned upon the emergence of the corresponding entity. Distributions (*U* or 𝒩) indicate variability in parameter values across entities. For normally distributed parameters (𝒩), the mean value is inherited from the parent population at its formation, enabling lineage-specific traits and evolutionary trajectories. Likewise, the number of genes in the genotype is inherited from the parent population, so the default values for each segment apply only to the initial populations of the founder species.

**Environment and its dynamics.**

The simulated environment was configured to provide a sufficiently large and heterogeneous space for population expansion, diversification and long-term eco-evolutionary dynamics. A grid of 1000×1000 regions ensured extensive spatial structure, while each region was characterized by 24 environmental properties. Although low compared to the complexity of real ecosystems, this number reflects the idea that organisms typically respond to a limited subset of environmental meaningful variables [99], balancing biological interpretability with computational feasibility. Each 8-bit property can assume 256 values, providing a sufficiently rich yet tractable spectrum of environmental conditions to drive selective differentiation. The probability of local environmental change per iteration (<2.5%) was set to induce continuous adaptive challenges without destabilizing the system. Large-scale restructuring events (i.e., region merging and splitting) were disabled to isolate the effects of localized environmental dynamics on eco-evolutionary outcomes.

**Genotype architecture.**

Following the same rationale, genotypes were designed to capture core biological functions using a compact representation that enables scalable simulation. Each gene was encoded as an 8-bit binary sequence, allowing 256 possible alleles per locus. Populations carried 36 phenotypic genes to support trait variability and adaptive potential. Two reproductive genes were used to compute interbreeding compatibility via Hamming distance, ensuring sufficient resolution to distinguish between closely related lineages. One niche gene defined each population’s ecological strategy, enabling representation of up to 256 discrete niche positions within the niche space. This setup allowed for functional and evolvable genomes while maintaining computational efficiency.

**Genetic variation and population processes.**

Building upon the genotype structure described above, mutation and gene-flow rates for phenotypic, reproductive, and niche genes were chosen within empirically supported ranges [100], enabling biologically plausible modulation of evolutionary dynamics. Phenotypic genes were assigned higher mutation rates ($\approx \;5\;\cdot \;10^{-4}$), reflecting their role in driving adaptive responses [84,101], while lower rates were used for reproductive ($\approx \;\;\mathrm{-1pt}10^{-4}$) and niche genes ($\approx \;\;\mathrm{-1pt}10^{-5}$) to model the slower evolution of reproductive isolation [84] and ecological tolerances [102]. Rather than using fixed values, allowing these parameters to vary across populations promoted heterogeneity in evolutionary strategies within the same simulation. This enabled, for instance, the coexistence of populations with extensive gene flow–favoring genetic homogenization and constraining divergence–and others with highly restricted gene flow–fostering local adaptation and isolation [103–105].

A similar rationale guided the parametrization of population-level processes. Splitting and migration rates varied across populations and were inherited upon lineage formation, representing a spectrum of life-history strategies ranging from sedentary to highly dispersive lineages [104,106]. This design supports the emergence of both localized persistence and demographic expansion within a single simulation.

**Phenotype construction.**

Although the number of phenotypic genes is reduced relative to biological organisms, the modular recombination, codon reuse and iterative transformations inherent to the GE-based GPM enable the emergence of complex phenotypic structures spanning multiple traits (see framework description for details). This mechanism enhances evolvability and innovation while keeping genome sizes manageable. Empirical evidence further supports that much of the variation in ecologically relevant traits can be explained by a limited set of genetic parameters [107,108], while developmental constraints and demographic stochasticity limit the effective phenotypic dimensionality subject to selection [36,109].

**Speciation and extinction.**

Reproductive divergence between conspecific populations was evaluated through the Hamming distance between their reproductive gene segments, with a species-specific threshold determining when divergence results in speciation. This allows reproductive isolation to emerge heterogeneously under a shared genomic architecture. Threshold values were selected to balance evolutionary stability and diversification potential, avoiding rapid speciation from minor genetic changes while still permitting divergence under certain genetic differentiation.

Extinction was modeled through two configurable–fitness-driven and ecological-interactions-driven–and one structural route (see framework description). Parameter values were chosen to prevent any single route from dominating the extinction dynamics. For example, the fitness threshold was set low to reflect lenient selection, eliminating only extremely poorly adapted populations. In combination, the three routes generate moderate but persistent ecological and evolutionary pressures, driving turnover and diversification without destabilizing system dynamics.

## Results and discussion

To explore how interactions among eco-evolutionary processes shape macroevolutionary patterns within the proposed framework, we conducted multiple independent forward simulations based on the general scenario outlined in the Methods. Each simulation ran for $5\;\times \;10^{6}$ iterations, enabling the observation of emergent dynamics across extended macroevolutionary timescales.

These proof-of-concept simulations were designed to examine how complex biodiversity patterns arise under idealized conditions–specifically, with no predefined selection pressures, unbiased initial conditions, and without imposed directional trends. To ensure a neutral starting point, the environmental properties of each region were randomly initialized, and a single founder species with a randomly assigned genotype was introduced in a randomly selected location. This neutral initialization prevented inherent biases in diversification or extinction, ensuring that observed evolutionary dynamics emerged solely from the modeled processes–i.e., genetic variation, ecological interactions, and environmental change.

### Starting the game: the challenge of establishing life

**Fig 2:**
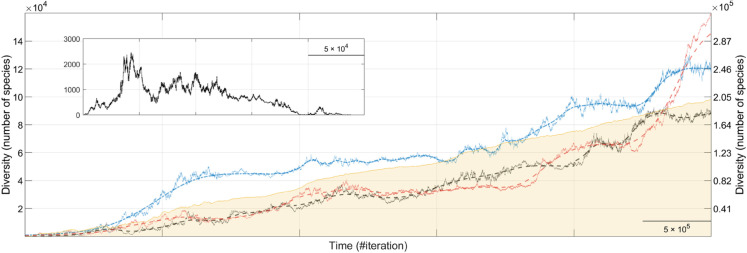
Temporal evolution of biodiversity. Representative biodiversity trajectories from simulations of the general evolutionary scenario. Dotted lines depict three individual simulations: blue and black illustrate the predominant saturating growth pattern (plotted on the left axis), while red represents the less frequent case of unbounded increase (plotted on the right axis). Solid lines show smoothed trends obtained using a Gaussian filter. The yellow shaded area marks the average biodiversity trajectory across 1,000 simulations, emphasizing the prevalence of saturating growth patterns, albeit smoothed by averaging. The inset shows an example simulation where life fails to establish in the long term. Note that iterations correspond to eco-evolutionary time steps rather than individual generations.

A first remarkable result of our simulations is that, in approximately half of the replicates, all life goes extinct early in the simulation, regardless of the initial population or species count. In some cases, this extinction is a direct consequence of random initialization: founder populations occasionally appear in regions where environmental conditions are so unfavorable that their adaptive capacity is insufficient, rendering them unviable from the outset and leading to rapid extinction. However, in other cases, populations initially manage to establish and diversify but still fail to persist in the long term (see inset in Fig 2). Note that in the interactive web application, this early fragility can be explored directly by enabling or disabling a temporary “survival boost”, which relaxes extinction-related processes during the first iterations and illustrates the difficulty of establishing life under full ecological pressure (see the web application for further details).

This high early extinction rate underscores the inherent challenges of sustaining viable populations in dynamic environments, where the conditions necessary for persistence and evolutionary diversification do not always arise. Newly originated species typically consist of only a few founder populations with limited genetic, phenotypic, and ecological variability. This narrow variation restricts their ability to cope with novel environmental conditions and fluctuations, making them highly susceptible to extinction. Only when sufficient diversity accumulates–through genetic mutation, ecological differentiation, or spatial expansion–can populations overcome the initial viability threshold and establish self-sustaining evolutionary trajectories.

These results emphasize that the emergence of biodiversity is not a deterministic outcome, but a contingent process shaped by the interplay of different eco-evolutionary factors and path-dependent dynamics. The very existence of nonzero biodiversity requires more than just environmental conditions compatible with the emergence and persistence of a material substratum (i.e., life), it depends crucially on the stochastic accumulation of diversity and the establishment of adaptive and diversification mechanisms that propel evolution forward. These requirements are particularly critical in the early stages of diversification, where small differences in initial conditions can decisively determine whether a lineage flourishes or collapses.

In the following sections, we focus our analyses on 1,000 simulations in which life successfully persisted and evolved, allowing macroevolutionary patterns to emerge.

### Dynamical patterns of biodiversity over time

#### Global biodiversity trajectories.

As a first step in understanding how biodiversity evolves in our simulations, we analyze global species richness trajectories across the entire environment and find that biodiversity exhibits a continuous increase over time. A more detailed inspection reveals two predominant diversification patterns: a biphasic saturating trajectory and, less frequently, a seemingly unbounded diversification trajectory.

In the saturating pattern, biodiversity growth slows down over time, reflecting constraints imposed by ecological interactions and niche space limitations (see “Emergent niche structuring and self-organization” section). Notably, this is the typical outcome produced under the “Balanced default conditions” preset in the web application. The pattern is characterized by alternating phases of diversity stability and gradual growth, punctuated by episodic bursts of rapid diversification of varying amplitudes (black and blue traces in Fig 2). These bursts primarily emerge in response to environmental changes and the dynamic evolution of ecological niches, prompting populations to adapt and diversify. In this regard, it is important to highlight how slow, gradual eco-evolutionary changes at lower levels shape rapid diversification bursts at the macroevolutionary level. This contrast reinforces the nonlinear relationship between microevolutionary mechanisms and macroevolutionary dynamics. This episodic diversification pattern aligns with macroevolutionary models proposing that evolution proceeds through long periods of relative stasis, interrupted by bursts of rapid radiation [110,111].

In contrast, in the unbounded biodiversity growth pattern (observed in approximately 4% of runs), species richness continues to increase throughout the simulation (red trace in Fig 2). These cases reflect conditions favoring rapid adaptive radiation or high ecological turnover, as exemplified by the “Unbounded biodiversity growth pattern and spatial constraints” preset in the web application, and enforced for illustrative purposes under the “Unlimited survival conditions” preset (see the web app for further details). This pattern resembles exponential diversification models proposed for certain taxonomic groups and geological periods, where global diversity expands without a fixed upper limit [112–114]. Further investigation is required to determine whether such unbounded patterns emerge from specific properties of the organisms’ GPMs, facilitating evolvability through the generation of novel phenotypic variants and adaptive solutions [74,75], or is a joint property emerging from a specific architecture in the network of species interactions.

**Fig 3:**
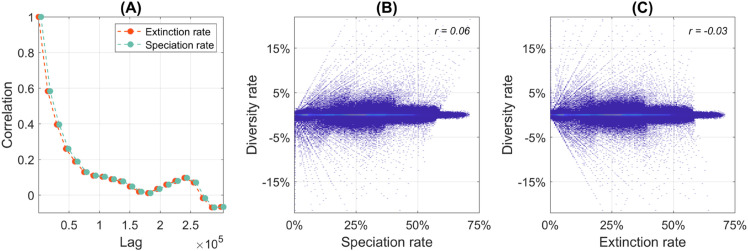
Correlation analysis of speciation, extinction and diversification rates per iteration. (A) Correlograms of speciation and extinction rates across different time lags in all the simulations, showing no significant autocorrelation at any interval. (B) Lack of temporal dependence between diversification and speciation rates, as indicated by the near-zero Pearson correlation. (C) Equivalent analysis for the extinction rate, confirming an absence of temporal dependence.

These two diversification patterns closely align with those inferred from empirical data. The saturating model (cf. [115], Fig 1b) has been widely used to describe long-term marine biodiversity trends, where diversity accumulation slows over geological timescales due to ecological and evolutionary constraints [115,116]. Conversely, the unbounded model (cf. [47], Figs 3B and 3C) has been associated with high ecological turnover scenarios, such as rapid post-extinction recoveries [78,112,117]. Indeed, these contrasting diversification dynamics have fueled long-standing debates over the mechanisms driving large-scale biodiversity trends, particularly regarding the biodiversity trajectory throughout the Phanerozoic. While some authors argue for continuous, nearly exponential diversification leading to historically unprecedented levels of biodiversity [113,118], others propose that diversity has remained near equilibrium for much of Earth’s history following a logistic growth pattern and fluctuating around ecological carrying capacities [119,120].

Our results suggest that this apparent dichotomy may instead reflect a probabilistic outcome of eco-evolutionary dynamics. On one hand, they suggest that saturating diversity growth is the most plausible long-term outcome under relatively stable eco-evolutionary conditions, supporting the idea that biodiversity accumulation is ultimately regulated by ecological interactions, environmental constraints, and evolutionary dynamics. On the other hand, the subset of simulations exhibiting seemingly unbounded biodiversity growth suggests that, under specific conditions, diversification can proceed at high rates for extended periods. This is consistent with biological scenarios in which rapid adaptive radiation follows major ecological opportunities [117,121]. In these cases, biodiversity expansion may initially appear exponential, but as niche availability becomes saturated or competitive interactions intensify, diversification rates slow, leading to a transition toward a more stable diversity equilibrium. A second dimension of this debate [118,122], which also emerges from our results, concerns contemporary ecosystems: have they already reached, or are they approaching, their maximum ecological capacity at local and/or global scales, or does diversification potential remain underexplored?

Given the coexistence of exponential, sublinear, and steady-state diversity dynamics within single evolutionary trajectories, it is tempting to speculate that speciation may itself be a self-organized process. That is, evolution may converge, through trial and error, on a speciation rate that optimally facilitates the long-term exploitation of a given environment. However, this hypothesis remains to be tested and warrants further empirical investigation.

#### Small-scale extinctions.

Superimposed on long-term trends, biodiversity trajectories exhibit small-scale stochastic fluctuations throughout the simulations (cf. individual time series in Fig 2). These fluctuations conceptually align with the process of *background extinction* [123], in which species that fail to adapt to localized environmental changes on short timescales are gradually eliminated, shaping long-term biodiversity dynamics [124].

In our simulations, these fluctuations emerge consistently as a macroevolutionary phenomenon, even in the absence of external environmental perturbations and during periods of apparent biodiversity stability. They result from the interplay of species competition, negative ecological interactions, niche shifts, and fluctuating selective pressures, which can destabilize populations even without major environmental change. Although most of these fluctuations lead to minor diversity losses, some intensify into transient but significant biodiversity declines, akin to temporary biodiversity contractions observed in the fossil record (cf. [125], Fig 1A; [126], Fig 3). This amplification of background extinction arises from the cascading propagation of localized species extinctions through ecological networks across different regions, occasionally triggering broader biodiversity declines that mark transitions to new biotic compositions.

These results highlight the role of fine-scale extinction events as a fundamental driver of macroevolutionary patterns, supporting the hypothesis that extinction risk is shaped not only by external abiotic environmental shifts but also by species interactions and emergent ecosystem-level dynamics [77,78,127]. More broadly, they reveal that extinction dynamics arise from the interplay of multiple underlying processes, rather than being dictated by a single dominant factor.

### Speciation-extinction balance and species turnover

To better understand how speciation and extinction shape biodiversity in our model, we analyze and compare their temporal evolution across long-term simulations. Unlike empirical macroevolutionary studies, which often rely on incomplete data (e.g., due to uneven preservation or taphonomic artifacts), our simulations track every relevant evolutionary event, providing a continuous, fine-grained record that transcends temporal and spatial snapshots. In particular, by recording the precise timing of species origination and extinction, we quantify their rates per iteration and examine their temporal correlation over evolutionary time.

We first analyze their self-correlation patterns. Similar to fossil diversification rates [128], our results reveal no significant autocorrelation at any time lag for either speciation or extinction rates (cf. Fig 3A and [96], Fig S1), indicating that their temporal fluctuations arise primarily from stochastic variation rather than intrinsic cyclical dynamics. Notably, neither the speciation rate (cf. Fig 3B) nor the extinction rate (cf. Fig 3C) exhibits a significant global correlation with biodiversity change per iteration. This lack of direct correlation suggests that short-term changes in biodiversity are not predominantly driven by fluctuations in one of these rates alone, but rather emerge from complex interplay between diversification and extinction processes.

**Fig 4:**
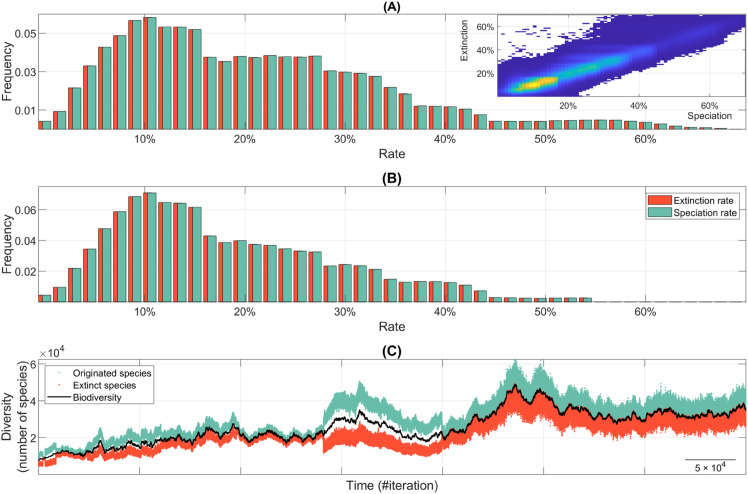
Fluctuating speciation-extinction dynamics and species turnover. (A) Histogram characterizing the distribution of speciation and extinction rates across iterations in all simulations of the general evolutionary scenario. Inset shows the general positive correlation between these rates. (B) Same analysis as in panel A, but excluding iterations where species richness drops below 3,000. This threshold, while arbitrary, provides a reasonable benchmark for assessing macroevolutionary dynamics after ecosystems have reached sufficient biodiversity levels. (C) Representative time series fragment showing phases of biodiversity stability, speciation bursts, and biodiversity declines. Cyan and red markers represent the number of newly originated and extinct species, respectively, at each iteration. Notably, periods of apparent biodiversity stasis often result from high species turnover where speciation and extinction balance out.

The comparison of the overall distribution of speciation and extinction rates across the 1,000 simulations reveals that species origination and extinction remain closely balanced, with gains and losses occurring at similar rates (Fig 4A). Correlation analyses further confirm this equilibrium, showing a strong positive relationship between both rates (see inset in Fig 4A). Specifically, the correlation coefficient across simulations is *r* = 0.89, indicating that episodes of diversification are frequently accompanied by increased extinction, and vice versa. This supports the notion of a short-term dynamic coupling between these antagonistic processes, which underlies the apparent equilibrium observed in long-term diversity patterns despite considerable temporal fluctuations in individual rates. An illustrative example of this counterbalancing dynamic is shown in Fig 4C. Similar equilibrium relationships, observed on geological timescales, have been reported in empirical studies based on fossil data (cf. [129], Fig 4).

**Fig 5:**
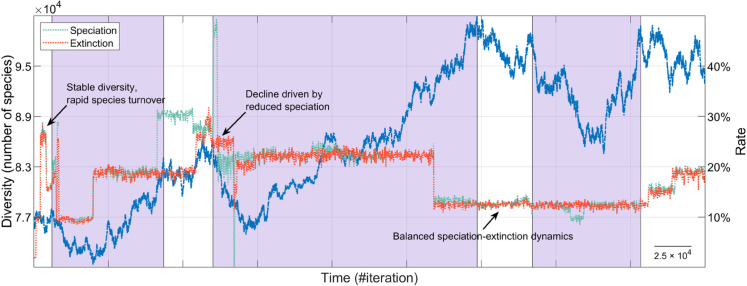
Lagged biodiversity recoveries. Fragment of the blue time series of Fig 2, showing three delayed recoveries following major extinction events (shaded regions). The corresponding speciation and extinction rates are plotted alongside. To better visualize trends, these rates are computed by averaging values over 200 consecutive iterations, reducing noise and highlighting overall dynamics. Consistent with previous results, the general pattern shows speciation and extinction closely following each other, with origination slightly exceeding extinction, driving the overall increase in diversity. This relationship shifts during periods of biodiversity decline. Notably, the figure illustrates cases where biodiversity loss is driven by reduced speciation rather than increased extinction (e.g., the event indicated by the arrow, where a prior burst of speciation reaching nearly 50% is followed by a sharp drop in speciation, while extinction rates remain nearly constant or even decrease). Likewise, some episodes of rapid diversification coincide with elevated extinction rates.

In our simulations, biodiversity time series often exhibit a lag of thousands of iterations between peaks of increasing and decreasing diversity (Fig 5A). This suggests that, after extinction events, new species gradually occupy vacant ecological niches until biodiversity recovers enough to sustain further slow diversification [130]. This pattern mirrors fossil record analyses (cf. [128], Fig 1a), and occurs not only after mass extinctions [117,131] but also following background extinction events, where extinction peaks precede subsequent diversification by approximately 10 million years [128]. Interestingly, our simulations also reveal cases where extinction peaks follow bursts of speciation, suggesting that rapid diversification can trigger runaway competition, ecosystem instability, and subsequent extinction waves [78].

A common explanation for delayed biological recoveries is a lag between extinction peaks and subsequent diversification peaks (cf. [128], Figs 1b and 1c). While this lagged speciation-extinction correlation is also observed in our simulations, it is not the primary driver of many delayed recoveries. As discussed above, the correlation between speciation and extinction rates typically remains high throughout the entire simulation. For instance, in the example shown in Fig 5, the global correlation coefficient is *r* = 0.92, indicating that these processes are generally well-balanced. Nevertheless, during specific phases, this correlation can drop. A notable example occurs during the second biodiversity decline in Fig 5, where the correlation coefficient decreases to *r* = 0.74, suggesting a partial and transient decoupling of these dynamics. A closer inspection of this decline reveals that it follows an extremely high speciation peak and, more interestingly, that biodiversity loss in this case is driven by persistently reduced speciation while extinction rate remains relatively stable. This pattern, frequently observed in our simulations, suggests that in many cases, recovery time is primarily determined by how long it takes for speciation rates to return to equilibrium with extinction rates, rather than by a particularly severe extinction rate. Instead, other biodiversity declines are characterized by abrupt waves of extinction followed by a swift compensatory increase in speciation, which maintains a stronger correlation between both processes throughout the recovery period. These findings suggest that different mechanisms may underlie biodiversity loss events [47], and that the predominance of one over another may shape the type, tempo, and mode of subsequent macroevolutionary recoveries.

A key insight from our simulations is the persistence of ongoing species turnover. As shown in Fig 4A, even under gradual environmental change speciation and extinction rates occasionally exceed 65% in individual iterations, with average rates falling within the 20%–25% range for over half of the iterations. To assess whether these extreme fluctuations are primarily an artifact of the early stages of the simulations, when overall diversity is still low, we repeat the analysis while excluding iterations in which species richness is below 3,000 (Fig 4B). While this constraint effectively reduces the magnitude of extreme speciation and extinction peaks to approximately 55%, these values remain strikingly high–comparable to turnover rates observed during major extinction events in the fossil record [132]. This reinforces the idea that high species turnover is an intrinsic feature of the evolutionary dynamics in our model, even during periods of apparent biodiversity stability. For instance, in Fig 4C and Fig 5, there are periods where, despite minimal net diversity change, speciation and extinction rates remain high, indicating a continuous cycle of species replacement at a fine temporal scale. In other words, in our simulations, significant evolutionary change may occur without major directional shifts in total diversity.

While phenotypic plasticity allows organisms to survive and adapt to moderate, transient environmental changes [133,134], recent evidence suggests that extinction risk increases when the rate of adaptive peak shifts outpaces both genetic and plastic adaptive capacities, ultimately leading to extinction [135]. Our analyses indicate that this is unlikely to be the primary driver of extinction in the general evolutionary scenario, where eco-evolutionary dynamics unfold at a relatively slow pace. However, when environmental change accelerates (see next section), this pattern of extinction becomes more pronounced.

The results discussed in this and the previous sections challenge traditional views of extinction, often conceptualized as a process of low background rates punctuated by occasional high-turnover events [136,137]. Instead, our findings support a more continuous and fine-grained extinction dynamic, where species turnover remains persistently high even during periods of apparent global biodiversity stability. This contradicts the assumption that long-term stability equates to ecological or evolutionary stasis. Thus, the apparent smoothness and directionality of long-term biodiversity trends–often resembling a quasi-steady-state dynamics–are not actually due to the absence of evolutionary change but rather to a delicate balance between speciation and extinction dynamics, which operate intensely and continuously in the background, yet on a finer scale. Therefore, global biodiversity trends may obscure substantial underlying dynamism, where species are continually replaced through trial-and-error evolutionary processes (see “Species longevity and extinction risk” section). This fine-scale turnover remains largely undetectable in fossil studies, where taxonomic stability can mask high extinction and speciation rates due to both sampling limitations and temporal averaging effects.

### Biodiversity dynamics under environmental shocks

Abrupt environmental perturbations–such as rapid climate shifts or asteroid impacts–have historically caused large-scale disruptions in biodiversity, often outpacing the rate of gradual environmental change [121,123]. While our general simulation scenario models continuous background environmental dynamics, we also tested the framework’s capacity to reproduce sudden biodiversity collapses by introducing short-term, high-intensity environmental disruptions (illustrated in the “Environmental shocks” preset of the web application). These global shocks were implemented as temporary increases in the rate of environmental change ($p_{region_{change}}$), simulating large-scale disturbances with controlled intensity and duration. The aim of these simulations was to evaluate whether the model could replicate one of the most fundamental macroevolutionary phenomena: mass extinction followed by ecological reorganization.

**Fig 6:**
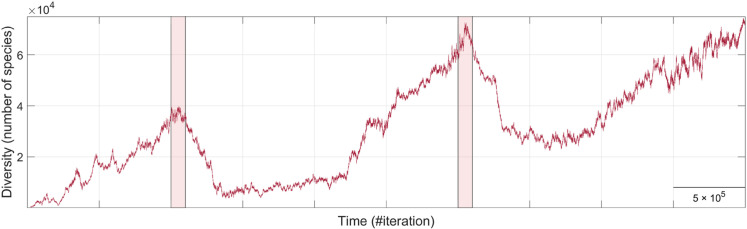
Biodiversity response to environmental shocks. At timesteps t=1,000,000 a.u. and t=3,000,000 a.u., a global environmental shock is simulated by setting $p_{region_{change}}=0.01$ for 100,000 iterations (shadowed regions). In response, biodiversity experiences a sharp decline of approximately 85% in the first event and 60% in the second event. Notably, biodiversity continues to decrease even after the perturbations cease, highlighting the cascading effects of species loss and the delayed nature of macroevolutionary recoveries.

As illustrated in Fig 6, these shocks trigger extinction events of varying magnitudes, resulting in rapid biodiversity loss. In the examples shown, total species richness declines by approximately 85% and 60%, respectively. The magnitude and temporal profile of these collapses are comparable to those inferred for Earth’s five major mass extinction events [132,138], highlighting the model’s potential to simulate abrupt ecological and evolutionary responses to large-scale environmental upheaval.

Notably, biodiversity continues to decline even after the perturbation ends. External shocks cause an abrupt increase in extinction rates, generating waves of species loss. As discussed in the previous section, these extinction cascades destabilize ecosystems and further reduce their capacity for adaptive recovery. Similar patterns have been inferred from the fossil record, where post-extinction diversification often occurs at slower rates than initial biodiversity loss [117,131]. Our simulations indicate that once species loss surpasses a critical threshold, the ecological conditions required for recovery become increasingly constrained, limiting the potential for biodiversity rebound. In extreme cases, recovery may be significantly delayed or even prevented (see below). These findings suggest that external shocks not only drive immediate biodiversity loss but also reconfigure macroevolutionary dynamics by reshaping species interactions, niche availability, and adaptive landscapes, ultimately influencing long-term diversification and extinction patterns.

Simulations with abrupt stressors also show a striking contrast between the initial difficulty of sustaining viable populations and the long-term resilience of well-established, mature communities. As noted earlier, in approximately half of the runs of the general scenario, all life goes extinct shortly after the simulation begins. This highlights the inherent challenge of crossing an initial viability threshold, where newly originated species, with limited genetic, phenotypic, and ecological variability–and thus limited adaptive capabilities–are particularly vulnerable to stochastic extinction. However, once ecosystems surpass this threshold and reach stable diversity levels, global extinction becomes exceedingly rare. While some regions experience complete local collapses–leading to the disappearance of all populations within them–biodiversity loss at the global scale only occurs when perturbations reach extreme levels, either due to the intensity of environmental shifts or the prolonged duration of stressors, pushing the system beyond a point of no return. This suggests a form of ecosystem inertia, where mature communities exhibit high resilience due to accumulated genetic and ecological diversity, buffering them against environmental fluctuations.

Interestingly, biodiversity resilience increases in simulations where environmental shocks are counterbalanced by greater adaptive potential. For instance, increasing mutation rates ($p_{mut_{p}}$) or reducing gene flow across populations ($p_{flow_{p}}$) in phenotypic genes enhances the ability of populations to track environmental changes more effectively, increasing their chances of persistence. These results support the hypothesis that many extinctions in Earth’s history may have resulted from scenarios in which the rate of adaptive peak shifting outpaced the ability of populations to adapt [135]. However, even under such favorable conditions, species that initially survive environmental disturbances may eventually fail to track ongoing selective pressures, leading to delayed extinction cascades and biodiversity collapse. This underscores the intricate interdependence of eco-evolutionary processes, where short-term survival does not necessarily guarantee long-term persistence.

In summary, our results suggest that extinction events, while disruptive, can also create ecological opportunities for diversification, ultimately reshaping macroevolutionary trajectories in unpredictable ways. The interplay between vulnerability and resilience observed in our simulations highlights the delicate balance governing post-disturbance recovery. While some ecosystems recover and diversify, others fail to reestablish, leading to prolonged biodiversity loss. This emphasizes the importance of identifying the factors that determine whether biodiversity rebounds or collapses following large-scale environmental perturbations. Bottom-up approaches that integrate eco-evolutionary interactions across temporal and spatial scales offer a powerful tool for exploring the mechanisms underlying these contrasting outcomes.

### Species longevity and extinction risk

The duration of a species, from its origin to its extinction, is a fundamental aspect of macroevolutionary dynamics [139]. Empirical analyses suggest that species exhibit a wide range of longevities, yet certain general patterns emerge across different taxonomic groups [123]. The general speciation-extinction coupling observed in our simulations (Fig 4) suggests that species longevity is closely linked to continuous species turnover. To further explore this relationship, we analyze species lifetime distributions across simulations.

**Fig 7:**
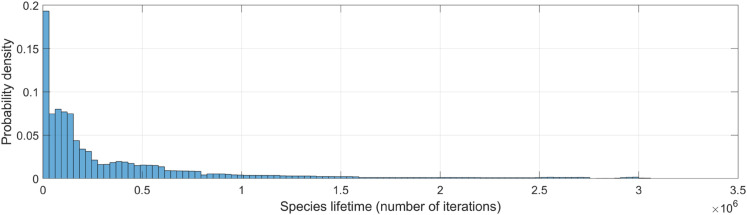
Histogram of species lifetime. Histogram depicting the lifespan of all species that emerge across the 1,000 simulations of the general evolutionary scenario. The distribution follows a long-tailed pattern, with most species being short-lived while only a few persist for extended periods, highlighting the continuous turnover dynamics shaping biodiversity.

Our simulations reveal a broad range of species longevities, yet their distribution is highly skewed, following a long-tailed pattern (Fig 7). Consistent with the challenges faced by initial founder species (see “Starting the game” section), most species are short-lived, while only a few persist for significantly longer periods. This pattern indicates that, although speciation is frequent, only a minority of lineages successfully avoid extinction for extended periods. This observation aligns with the general expectation that species persistence is inherently limited, as suggested in empirical studies reporting average species longevities on the order of millions of years [123,129].

A potential explanation for the skewed long-tailed lifespan distribution observed in our simulations is that evolutionary dynamics operate through an extensive continuous trial-and-error process at a fine-grained scale, where most evolutionary “experiments” fail to persist, and only a small fraction succeed and establish long-term lineages. This persistent species turnover suggests that evolution proceeds through the recurrent exploration of ecological opportunities, with biodiversity being maintained not by prolonged stability, but by the constant replacement of species. This aligns with models in which macroevolutionary dynamics emerge from high lineage turnover rather than deterministic trends toward long-term persistence of a few dominant taxa [51]. Our findings reinforce this perspective, emphasizing that high speciation rates do not necessarily lead to sustained biodiversity growth, as most newly arising species are short-lived.

These findings could have important implications for interpreting paleo-diversity trends. Fossil-based diversity reconstructions often rely on genus- or family-level data due to the incompleteness of the species-level fossil record. If most species are short-lived, their likelihood of being preserved in the fossil record decreases, leading to a potential overrepresentation of longer-lived taxa in paleontological datasets. This bias could skew macroevolutionary analyses, underestimating true species turnover rates and leading to an apparent, but misleading, perception of long-term biodiversity stability. Consequently, interpretations of diversification rates and extinction selectivity in deep time may need to account for the possible underrepresentation of ephemeral lineages.

### Emergent niche structuring and self-organization

The spatiotemporal organization of ecological niches within coevolving communities is a fundamental driver of biodiversity patterns and ecosystem stability. In real-world ecosystems species are distributed along environmental gradients, forming nearly continuous niche occupancy patterns [140]. This structured distribution enhances resource utilization efficiency and facilitates species coexistence by reducing competitive exclusion [141].

To assess whether similar patterns emerge in our simulations, we analyze the niche positions of populations coexisting in each individual region at the end of all simulations. Note that, in the general simulation scenario, niche position is encoded by a single 8-bit niche gene, allowing for 256 distinct ecological niches in each region (from 0 to 255). We find that in 74% of the final regions, all 256 available niches are occupied, meaning that, at that precise spatiotemporal context, at least one population is present in every niche position. To test the robustness of this occupancy pattern under expanded niche availability, we perform an additional set of 100 simulations using genotypes with expanded niche-encoding capacity, allowing niche positions to range from 0 to 65,535. The simulation time is also extended to allow populations to evolve and explore the broader niche range. Consistently, the niche occupancy gradient persists, with 58% of the final regions exhibiting full occupation of all 65,536 available niches.

**Fig 8:**
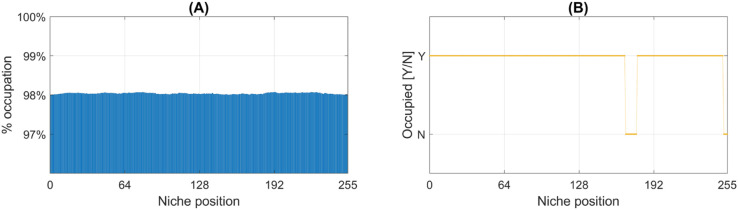
Niche gradient occupancy pattern. (A) Histogram showing the proportion of regions in which each ecological niche position (0–255) is occupied at the end of the 1,000 simulations. A niche is considered occupied in a region when at least one population is present at the corresponding niche position. (B) Illustrative example of observed discontinuities in the niche gradient occupancy pattern.

To further examine how populations distribute in regions that lack full niche occupancy in the final state of the general scenario simulations, we inspect which specific niche positions remain unoccupied. The most common situation is that fewer than 2% niches remain vacant. In other words, even when full niche occupancy is not reached in a given region, the overall niche distribution remains nearly complete. To quantify this, we analyze the occupancy frequency of each individual niche across all final regions (Fig 8A). We find that, for each specific niche position (0–255), at least one population is present in almost 98% of the analyzed regions. The few vacancies observed likely reflect brief, transient intervals between a niche being vacated and subsequently reoccupied. In these cases, populations and species with similar ecological requirements cluster together, forming continuous distributions along the niche gradient (e.g., see Fig 8B). Deviations from the described patterns are rare, occurring in fewer than 1% of regions.

These results indicate that the niche gradient occupancy pattern emerges spontaneously as a self-organizing process contributing to ecosystem stability, without requiring predefined niche structuring rules. This dynamic mechanism promotes the filling of all available niches, with vacant niches appearing only temporarily due to local extinctions or niche shifts. As a result, populations distribute within the available niche space in a way that not only aligns with their physiological constraints but also minimizes competitive overlap.

Beyond niche continuity, a detailed analysis of community composition reveals that, as biodiversity increases, competition and selective pressures promote the coexistence of broad-niche generalists and narrow-niche specialists, in a dynamic balance that emerges as a self-organizing property of the system. This result aligns with theoretical predictions that species evolution can give rise to a bimodal niche overlap distribution, facilitating the coexistence of both specialist and generalist species [142]. Furthermore, we find that as species richness rises, the proportion of specialists tends to increase, while generalists become less dominant. This pattern is consistent with empirical observations suggesting that highly diverse communities often support greater niche specialization [143]. However, our results also highlight that this balance fluctuates over evolutionary time, influenced by environmental variability and ecological interactions.

Another remarkable insight derived from a closer analysis of niche dynamics concerns the idea of *potentiality*, i.e., the distinction between a species’ full ecological potential and its actual distributions in a given spatiotemporal context. In our simulations, populations of a species do not always occupy a single fixed niche. Instead, they may be distributed across significantly different positions within the niche space over both space and time, reflecting responses to varying ecological conditions and realized selective pressures. For instance, in the final state of the simulations, we identify multiple species whose aggregated populations nearly span the entire available niche space, or even all positions (0–255). However, at the population level, the pattern is markedly different, with individual populations constrained within narrow, seemingly distant fundamental niche positions (e.g., 3–6, 126, and 217–228). This contrast suggests that local sympatric adaptations–likely aided by phenotypic plasticity–drive significant ecological differentiation even within species, rather than all populations expressing the full range of conditions that the species could *potentially* inhabit [99]. Likewise, a population’s realized niche is not static but shifts considerably over its evolutionary trajectory, from emergence to extinction. These findings underscore the role of intraspecific variability in sustaining species persistence and contributing to the overall stability of ecological communities. They also suggest that species exist within a dynamic landscape of ecological opportunities, where their realized niches are continuously created and reshaped by eco-evolutionary interactions, rather than being passively occupied based on intrinsic adaptive potential alone.

Overall, the findings discussed in this section reinforce the idea that large-scale ecological patterns can emerge from local eco-evolutionary processes without requiring explicit high-level constraints or organizing principles. The spontaneous structuring of niches, the emergence and coexistence of generalists and specialists, and the observed fluctuations in community composition suggest that macroecological patterns largely arise as emergent properties of eco-evolutionary dynamics rather than as direct consequences of individual adaptive strategies, as is often assumed. Understanding these self-organizing processes would provide new insights into the mechanisms shaping biodiversity patterns across ecological and evolutionary scales.

### Genetic drift as emergent process

Genetic drift, a fundamental microevolutionary mechanism, refers to stochastic fluctuations in allele frequencies due to random sampling effects [144]. Although genetic drift is not explicitly modeled in the framework, we assess whether it emerges naturally from the inherent stochasticity in the mutation-selection sub-algorithm. Specifically, we examine whether neutral genetic variation follows characteristic drift patterns, evolving independently of selective pressures.

To investigate this, we analyze the evolution of phenotypic gene frequencies within coexisting populations of the same species over generations. These genes fall into three categories: (i) fitness-influencing genes, which contribute to trait formation under selection; (ii) expressed but non-fitness-influencing genes, which participate in genotype-to-phenotype mapping but do not directly affect fitness; and (iii) non-expressed genes, which remain adaptively neutral. Since non-expressed genes experience no direct selection, they provide an ideal case for detecting stochastic genetic drift.

**Fig 9:**
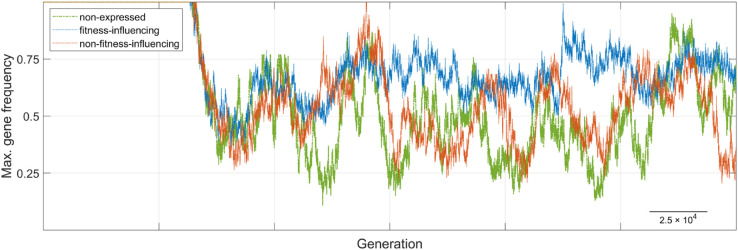
Evolution of phenotypic gene frequencies. Temporal dynamics of the most frequent allele value for three types of phenotypic genes within populations of a representative species during the first 300,000 iterations after originating from the initial founder population: non-expressed genes, which are selectively neutral and serve as indicators of genetic drift; fitness-influencing genes, which are subject to selection; and non-fitness-influencing genes, which participate in GPM but do not directly affect fitness.

The green trace in Fig 9 illustrates how the frequency of non-expressed genes of a representative species evolves over generations. As expected, the founder population starts with no genetic variability, but variation accumulates over time due to mutation. Consequently, the maximum frequency of any given “allele” gradually decreases as diversity increases. However, this process does not lead to a uniform distribution of all possible alleles across the species’ genotype. Instead, we observe a minimum allele frequency peak around 20%, indicating a relatively high degree of fixation (note that in our simulations each gene can take 256 possible values), albeit not complete. Eventually, some non-expressed genes reach near-complete fixation (∼100% frequency) during periods of population coexistence, indicating pervasive genetic drift across populations.

The stochastic nature of genetic drift is further reflected in the pronounced and erratic fluctuations of non-expressed gene frequencies over time. Similar fluctuations extend to expressed non-fitness-influencing genes (red trace), as they are not directly constrained by selection. In contrast, while fitness-influencing genes (blue trace) also experience genetic drift, their fluctuations are modulated by selection, which favors alleles that enhance fitness and leads to more predictable variation patterns.

In summary, our simulations show that genetic drift naturally emerges from the stochasticity of the mutation-selection process, causing allele frequency fluctuations even without explicit modeling and effectively contributing to fine-scale genetic variation. These random changes, in turn, interact with selection, gene flow, and mutation to shape large-scale evolutionary patterns.

## Conclusions

Understanding the underlying causes that generate and shape biodiversity over time and space remains a central challenge in evolutionary biology. This work presents a flexible and generalist simulation framework that integrates stochastic genetic dynamics, population dynamics, spatially structured ecological interactions and environmental change to explore macroevolutionary dynamics from a bottom-up, process-explicit perspective.

Through a series of proof-of-concept simulations, we show that the framework reproduces a wide range of macroevolutionary patterns, including saturating and exponential-like diversification, episodic bursts of speciation, background and mass extinctions, long-tailed species longevity distributions and dynamic niche occupancy. These patterns emerge from the interplay of fundamental microevolutionary, ecological and stochastic processes, without requiring artificially predefined trajectories or externally imposed dynamic, which illustrates the model capacity to bridge micro- and macroevolutionary scales. The presence of self-similarity across scales and emergent non-linear feedbacks suggests that the system exhibits dynamics comparable to complex systems near the “edge of chaos”, where small perturbations can lead to disproportionate effects [145]. These findings support the view that macroevolutionary patterns cannot be reduced to isolated causal factors [113], and contribute to the long-standing debate over the relationship between micro- and macroevolution [17,21,22].

Although direct quantitative comparisons with empirical data are inherently limited by scale mismatches, unit definitions and data resolution, the model provides a heuristic platform for investigating the generative logic of biodiversity dynamics. Its open-endedness–a rarely achieved feature in eco-evolutionary modeling–along with its modularity, functional representation of phenotypes and multiscale organization, position it as a complementary tool to existing approaches. Beyond its theoretical contributions, the framework can also support hypothesis testing and educational exploration through user-defined scenarios and parameter manipulation.

## Supporting information

S1 Fig Illustrative example of GE-based GPM. The mapping process begins with the initial non-terminal symbol of the grammar, <*S*>, and recursively continues, guided by the 2-bit codons encoded in the phenotypic segment (represented in decimal between brackets for clarity), until all symbols become terminal. Note that not all phenotypic genes contribute to the mapping, reflecting the modular and selective nature of biological processes.
(TIFF)

S1 Text Supplementary appendices. S1. Detailed information and illustrative examples of how the macroevolutionary pathway of niche evolution operates.
(PDF)
