# Supplementary material for: Simulating macroevolutionary trends and open-ended evolution with a novel mechanistic multi-level approach
Source: PLoS One. 2025 Nov 6;20(11):e0335033. doi: 10.1371/journal.pone.0335033 (PMC12591426; doi:10.1371/journal.pone.0335033)
Supplement: S1 Text — S1. Detailed information and illustrative examples of how the macroevolutionary pathway of niche evolution operates. (PDF) [file pone.0335033.s002.pdf]

Supporting information for:

Simulating macroevolutionary trends and open-ended evolution with a novel mechanistic multi-level approach

Roberto Latorre[1], Miguel Brun-Usan[2,3], Gloria Fernández-Lázaro[4]

[1] Grupo de Neurocomputación Biológica, Dpto. de Ingeniería Informática, Escuela Politécnica Superior, Universidad Autónoma de Madrid, 28049, Madrid, Spain

[2] Dpto. de Biología, Unidad de Paleontología, Facultad de Ciencias and Centre for the Integration in Paleobiology (CIPb-UAM), Universidad Autónoma de Madrid, 28049, Madrid Spain

[3] Centro Andaluz de Biología del Desarrollo (CABD), Universidad Pablo de Olavide-CSIC, 41013, Seville, Spain.

[4] Dpto. de Psicología Biológica y de la Salud, Facultad de Psicología, Universidad Autónoma de Madrid, 28049, Madrid, Spain

Table of contents:

Ecological niche macroevolutionary pathway	Page 1

Ecological niche macroevolutionary pathway

The macroevolutionary pathway shifting populations toward new ecological niches is driven by ecological pressures. To quantitatively assess the selective pressure experienced by a population at a given moment, we compute a *net ecological impact* (NEI) considering both competitive and non-competitive interactions within its niche.

On the one hand, the degree of niche overlap, calculated as the shared range of the niche divided by the total range, allows us to quantify the intensity of competition among populations. To account for the possibility that strong adaptations balance intense competitions due to high niche overlap, we consider a weighted degree of niche overlap where the difference between the fitness indicators of the competing populations is introduced as correction factor. On the other hand, non-competitive interactions are weighted equally as a function of a general simulation parameter (w_{rel}). Positive and negative interactions are assigned opposite signs to distinguish their effects. Therefore, the net ecological impact on a population A in each time step of the simulation is computed as indicated in the main text:

$$NEI_A = \sum_{i \neq A} \left[\left(L_{A,i} \cdot (1 - F_{A,i}) \right) + W_{A,i} \right]$$

where $L_{A,i}$, $F_{A,i}$ and $W_{A,i}$ denote the degree of niche overlap, the fitness difference, and the weight of non-competitive impacts between populations A and i, respectively.

To illustrate the macroevolutionary pathway, let consider four coexisting populations and analyze the potential ecological relationships of population A:

- Population A has a niche range of 10-20 and a fitness indicator of 1.0, indicating optimal adaptation.
- Population B has a niche range of 12-250 and a fitness indicator of 0.2, indicating poor adaptation.
- Population C has a niche range of 18-88 and a fitness indicator of 0.9, indicating high adaptation.
- Population D has a niche range of 50-51 and a fitness indicator of 0.75, indicating moderate-high adaptation. Additionally, it has a facilitative relationship with population A (assuming $w_{rel} = 0.5$).

Population A competes with population B, with an 80% niche overlap between them ((20 - 12) / (20 - 10)). Similarly, it also competes with population C, with a 20% niche overlap ((20 - 18) / (20 - 10)). These niche overlaps suggest significant competition with B and potential coexistence with minimal competition from C. Populations A and D do not compete through niche overlap. Instead, they interact through facilitation, resulting in a positive impact of 0.5 on A. If we consider only niche overlap and not fitness, the net ecological impact for population A is -0.5 (-0.8 - 0.2 + 0.5). However, factoring in fitness indicators changes the competitive pressure on population A. As it is better adapted to the environment than populations B and C, the competitive pressure is lower than expected based on niche overlap alone. The calculations yield 0.16 (0.8 * (1.0 - (1.0 - 0.2))) and 0.18 (0.2 * (1.0 - (1.0 - 0.9))) as the weighted degree of niche overlap from B and C, respectively. This results in a net ecological impact of 0.16, pointing out a favorable balance of interactions and indicating that population A is well-adapted to its current niche. The fitness correction factor plays a significant role in this example. Excluding it would force population A to evolve (cf. 0.16 vs. -0.5 in the net ecological impact). Additionally, this correction influences the ecosystem dynamics, with populations B and C having net ecological impacts of -0.62 and -0.34, respectively. Despite population B's apparent advantage in being less specialized and having less

niche overlap with other populations, it must evolve or likely face extinction due to the interplay of fitness and niche overlap.
